# Comparative Proteomic Characterization of Ventral Hippocampus in Susceptible and Resilient Rats Subjected to Chronic Unpredictable Stress

**DOI:** 10.3389/fnins.2021.675430

**Published:** 2021-06-17

**Authors:** Yani Zhang, Xiaoling Zhang, Nuo Liu, Siyu Ren, Congyuan Xia, Xiong Yang, Yuxia Lou, Huiqin Wang, Ningning Zhang, Xu Yan, Zhao Zhang, Yi Zhang, Zhenzhen Wang, Naihong Chen

**Affiliations:** ^1^State Key Laboratory of Bioactive Substances and Function of Natural Medicines, Institute of Materia Medica and Neuroscience Center, Chinese Academy Medical Sciences and Peking Union Medical College, Beijing, China; ^2^Institute of Clinical Pharmacology and Science and Technology Innovation Center, Guangzhou University of Chinese Medicine, Guangzhou, China; ^3^Department of Anatomy, School of Chinese Medicine, Beijing University of Chinese Medicine, Beijing, China

**Keywords:** chronic unpredictable stress, depression, label-free quantitative proteomics, stress-resilient, stress-susceptible

## Abstract

Chronic stress is an essential factor leading to depression. However, there exist individual differences in people exposed to the same stressful stimuli. Some people display negative psychology and behavior, while others are normal. Given the importance of individual difference, finding differentially expressed proteins in stress-resistant and stress-susceptible groups has great significance for the study of pathogenesis and treatment of depression. In this study, stress-susceptible rats and stress-resilient rats were first distinguished by sucrose preference test. These stress-susceptible rats also displayed depression-like behaviors in forced swimming test and open field test. Then, we employed label-free quantitative proteomics to analyze proteins in the ventral hippocampus. There were 4,848 proteins totally identified. Based on statistical analysis, we found 276 differentially expressed proteins. Bioinformatics analysis revealed that the biological processes of these differential proteins were related to mitochondrion organization, protein localization, coenzyme metabolic process, cerebral cortex tangential migration, vesicle-mediated transport, and so on. The KEGG pathways were mainly involved in metabolic pathways, axon guidance, autophagy, and tight junction. Furthermore, we ultimately found 20 stress-susceptible proteins and two stress-resilient proteins. These stress-related proteins could not only be potential biomarkers for depression diagnosis but also contribute to finding new therapeutic targets and providing personalized medicine.

## Introduction

Depression is a psychiatric disorder with high rates of morbidity and mortality. The main clinical features of this prevalent disorder are low mood, slow thinking, declined cognitive ability, anhedonia, and other somatic symptoms (Smith, 2014; Rotenstein et al., 2016; Malhi and Mann, 2018; Fontanella et al., 2020). The World Health Organization ranks depression as the single largest contributor to global disability and estimates that depression will be the second-largest disease worldwide in 2020. The causes and pathophysiology of depression have been preliminarily recognized (Grace, 2016; Ménard et al., 2016; Ferrer et al., 2020; Li, 2020; Prakash et al., 2020). However, no single hypothesis can explain all aspects of depression.

Nowadays, increasing studies have suggested that depression is closely related to environmental factors (Kessler, 1997; Hammen, 2018). All kinds of stressful events may lead to depression, such as chronic illness, divorce, poverty, unemployment, childhood maltreatment, bereavement, and so on (Brown et al., 2013; Gonda et al., 2016; Kim et al., 2017). Chronic exposure to stress leads to depression-like behaviors in rodents associated with the hypothalamic-pituitary-adrenal (HPA) axis imbalance, neuroinflammation, and other mechanisms (Barnum et al., 2012; Mahar et al., 2014; Dandekar et al., 2019). However, some individuals exposed to the same degree of stress without appearing depressive symptoms. Stress resilience (SR) is a phenomenon wherein the individual can maintain normal psychological and physiological functions under acute or more persistent chronic stress (Brachman et al., 2016; Han and Nestler, 2017). The occurrence of SR is not only associated with the lack of stress sensitivity but also is involved in the process of physiological and psychological adaptation. Strengthening molecular mechanism research of different stress phenotypes is vital in discovering the biomarkers and pathogenesis of depression and promoting studies on antidepressant drugs (Menard et al., 2017; Carboni et al., 2018).

The hippocampus is an important structure of the limbic system, closely related to emotion, learning, and memory. Researches on the pathology of depression show neuroplasticity changes in the hippocampus, including hippocampal neuron atrophy, decreased hippocampal volume, new neuron loss, and decreased dendritic spine density (Vythilingam et al., 2004; Abercrombie et al., 2011; Anacker et al., 2013; Dioli et al., 2019). With chronic exposure to stress, the hippocampus participated in the negative feedback regulation of the HPA axis (Wu et al., 2013). Recent findings suggest that the susceptibility of depression symptoms is related to the hippocampus (Webster et al., 2002; Bagot et al., 2016; Alves et al., 2017; Bluett et al., 2017; Zhang et al., 2019). The dorsal hippocampus (DHIP) is related to the regulation of cognitive function, and the ventral hippocampus (VHIP) is associated with anxious and depression-like behaviors, including response to stress and emotions. Therefore, scientists focused on the function research in VHIP (Fanselow and Dong, 2010; Bagot et al., 2015).

The occurrence and progression of depression involve the coordinated changes in multiple signaling pathways in cells (Zheng et al., 2016; Ogyu et al., 2018). Proteomics focuses on the large-scale analysis of overall proteins, have an advantage in molecular profiling, biomarker identification, complex biochemical system characterization, and pathophysiological process inspection (Comes et al., 2018; Gadad et al., 2018). Chronic unpredictable stress (CUS) procedure was employed to induce anhedonia, a core symptom of depression, which allows investigators to segregate susceptible and resilient rats (Wohleb et al., 2018; Zhang et al., 2020). We utilized label-free technology coupled with mass spectrometry to analyze proteins in VHIP. Then, differentially expressed proteins were screened to analyze the bioinformatics further. We aimed at finding the differential proteins relevant to stress susceptibility and SR, revealing the pathogenesis of depression at the cellular level and identifying more informative biomarkers of depression, which will aid the design and development of improved treatments and better health assessment.

## Materials and Methods

### Animals

Eight-week-old male Wistar rats (Vital River Laboratory, Beijing, China) were exposed to a 12-h dark/12-h light cycle at 25 ± 2°C temperature and 55 ± 5% humidity with food and water *ad libitum*. All the rats were of specific pathogen-free grade and weighing 180–200 g. All procedures were executed in compliance with the National Institutes of Health Guidelines for the Care and Use of Laboratory Animals. Protocols of animal experimentation were approved by the Animal Care Committee of the Peking Union Medical College and Chinese Academy of Medical Sciences.

### Chronic Unpredictable Stress Procedure

The CUS procedure was based on our previous experiment with some modifications (Chen et al., 2017; Jin et al., 2017). All applied stressors are shown in Supplementary Table 1. The researches show that the sucrose intake in CUS-exposed rats corresponded to different stress responses (Henningsen et al., 2012; Han et al., 2015). Sucrose preference test (SPT) was conducted before CUS treatment. These rats were excluded to avoid experimental errors, which had low sucrose intakes (lower than 8.5 g), or high standard deviation (SD) of sucrose intakes compared with average SD (more than two times). According to the baseline of sucrose, the rats were randomly divided into different groups and exposed to various scheduled stressors for 8 weeks to induce depression-like behavior.

**FIGURE 1:**
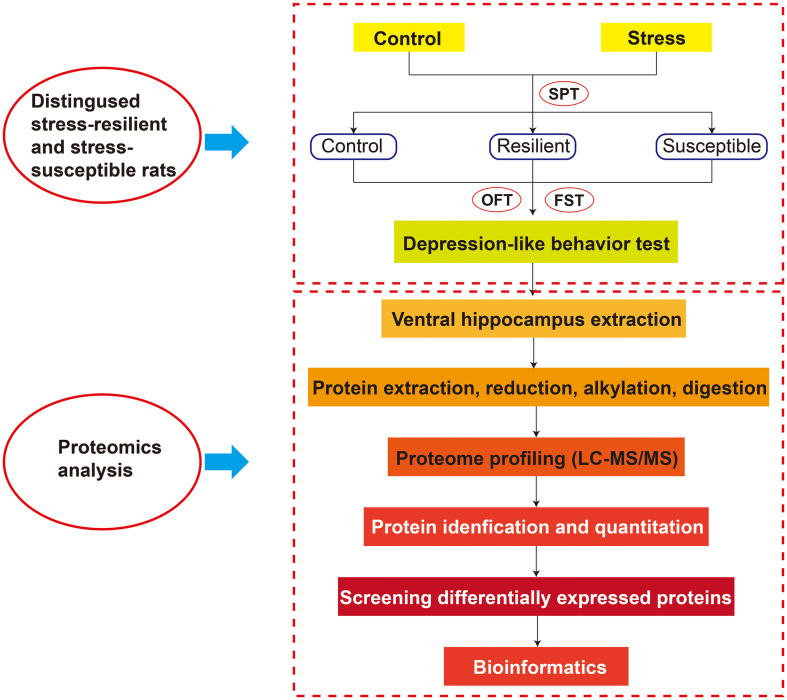
The whole experimental design.

After an 8-week CUS treatment, these rats were divided into two stress subtypes based on the results of SPT. These CUS-exposed rats were defined as stress-susceptible rats when their sucrose intake reduction was more than 30%, whereas those rats were defined as stress-resilient rats when their sucrose intake was no significant difference with baseline. At the same time, we employed open field test (OFT) and forced swimming test (FST) to evaluate the depression-like behavior. Behavioral test procedures are shown in the Supplementary Material. The whole experimental design is shown in Figure 1. For the post behavior test, the VHIP tissues were collected for label-free quantitative proteomics analysis.

### Protein Extraction and Trypsin Digestion

The protocols of protein extraction, reduction, alkylation, digestion, and fractionation were carried out as previously described with some adjustments (Chen et al., 2017). For protein extraction, rat VHIP tissue was homogenized in cold lysis buffer containing protease inhibitor cocktail; then, the samples were sonicated and centrifuged at 13,200 × rpm for 5 min at 4°C. We measured the protein concentration by the Qubit fluorescent protein quantification cation kit (Invitrogen, Carlsbad, CA, United States). Proteins were reduced at 45°C for 30 min by using dithiothreitol. For protein alkylation, 500 mM iodoacetamide was utilized, and the alkylation processing was conducted in the dark for 60 min at room temperature. Finally, the trypsin was used to digest proteins at a ratio of 1:50 at 37°C for 12 h. We collected these peptides and stored them at -80°C for further protein analysis.

### Liquid Chromatography-Tandem Mass Spectrometry

Samples were detected by Orbitrap Fusion Lumos mass spectrometers (Thermo Fisher Scientific, Rockford, IL, United States) coupled with an Easy-nLC 1000 nanoflow LC system (Thermo Fisher Scientific, Cambridge, MA, United States). Reverse-phase chromatography was performed to separate the digested proteins. First, the dried peptides were re-dissolved in mobile phase A (0.1% formic acid aqueous solution), where the mobile phase B was 0.1% formic acid in acetonitrile. Then these peptide samples were loaded to a homemade trap column (0.10 mm × 20 mm, 3 μm; Dr. Maisch, Ammerbuch, Germany) and further separated on a silica column (0.15 mm × 300 mm, 1.9 μm; Dr. Maisch, Ammerbuch, Germany). Gradient elution program started with 5% B, and gradually raised to 35% B within 141 min, then quickly raised to 95% B in 1 min, and ended with 95% B lasting for 9 min. We controlled the flow rate at 600 nl/min and the column temperature at 60°C.

The peptides were analyzed by Orbitrap Fusion Lumos mass spectrometry in data-dependent acquisition mode (Thermo Fisher Scientific, Rockford, IL, United States). Automatic gain control (AGC) targets for full-scan MS spectra were 5 × 10^5^ ions with a maximum injection time of 50 ms; AGC target value for MS/MS scans was 5 × 10^3^ charges with 35 ms. Under top-speed mode, the most intense ions were selected to be isolated in Quadrupole with a 1.6-m/z window. Normalized collision energy was set as 32% to fragment the ions, and the dynamic exclusion time was 25 s.

### Data Analysis

The raw data files were processed by Proteome Discovery 2.2 (Thermo Fisher Scientific, Rockford, IL, United States). We employed Mascot Server (www.matrixscience.com, Matrix Science, London, United Kingdom) for protein identification and quantitation based on UniProt KB protein database.^1^ The search parameters are shown as follows: The enzyme was trypsin, the value of maximum missed cleavages was 2, the fixed modification was carbamidomethyl (C), the value of peptide mass tolerance was ±15 ppm, fragment mass tolerance was 20 mmu, peptide confidence was high, the peptide length was set as more than 6, and the false discovery rate was set to 0.01. Differences among the three groups were analyzed by one-way ANOVA or Student’s *t-*test followed by LSD correction. The standard of statistical significance was set at *P* < 0.05. All experimental data were presented as mean ± SD. The statistical work was carried out using SPSS 11.5 statistic software (SPSS, Chicago, IL, United States).

## Results

### Depression-Like Behavior Assessment

**FIGURE 2:**
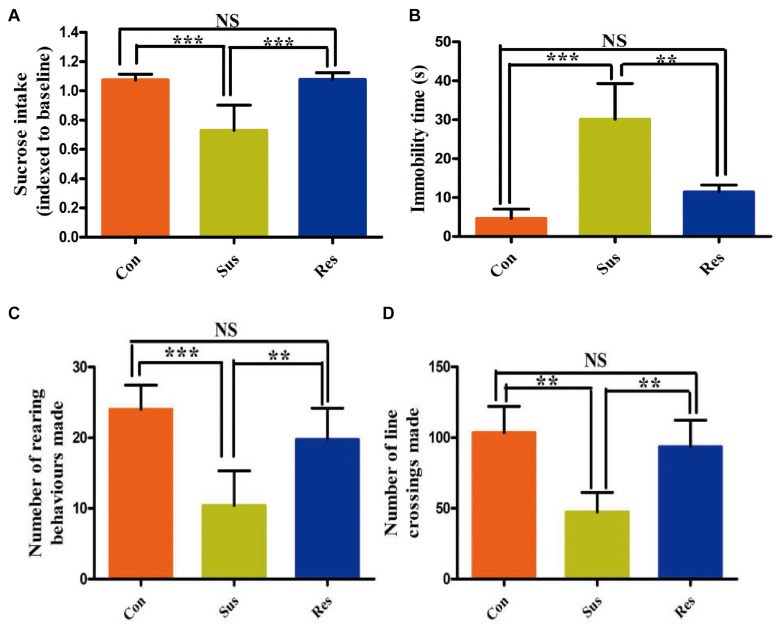
Chronic unpredictable stress (CUS) treatment induced phenotypes associated with stress susceptibility and stress resilience. **(A–D)** The results of sucrose preference test (SPT), forced swimming test (FST), and open field test (OFT). All data were shown as mean ± SD. ***P < 0.001; **P < 0.01. NS, no significant differences; Con, control; Sus, stress susceptible; Res, stress resilient.

In this study, sucrose consumption was employed to evaluate anhedonia degree of CUS-exposed rats (Figure 2A). After an 8-week CUS stimuli, stress-susceptible rats displayed a significant reduction in sucrose intake, and stress-resilient rats showed no significant changes in sucrose intake compared with control group. FST can reflect the desperate and helpless behavior of stress-induced rats. Our results showed that the immobility time was significantly increased in stress-susceptible rats compared with control group and stress-resilient group (Figure 2B). In OFT, the number of rearing and line-crossing behavior per 5 min showed a significant reduction in stress-susceptible rats compared with controls (Figures 2C,D). However, stress-resilient rats showed no significant difference in all behavior test results compared with control group.

### Protein Identification and Quantitation

A total of 4,848 proteins in VHIP were identified by label-free quantitative proteomics. Then according to fold change >1.5, *P*-value <0.05 or fold change 0.667, and *P*-value <0.05, we preliminarily screened differentially expressed proteins (Supplementary Tables 2–4). We discovered that 48 proteins were upregulated and 162 proteins were downregulated in stress-susceptible versus control group. There were 56 upregulated proteins, and 93 downregulated proteins in the stress-resilient versus control group. Meanwhile, compared with the stress-resilient group, 95 proteins showed a significant difference in the stress-susceptible group (Table 1).

**TABLE 1:** Statistics of differentially expressed proteins.

Comparisons	Upregulated	Downregulated
Sus vs. Ctrl	48	162
Res vs. Ctrl	56	93
Sus vs. Res	29	66

*Sus, stress-susceptible; Res, stress-resilient; Ctrl, control.*

### Differential Proteins Analysis in Chronic Unpredictable Stress-Exposed Rats

**FIGURE 3:**
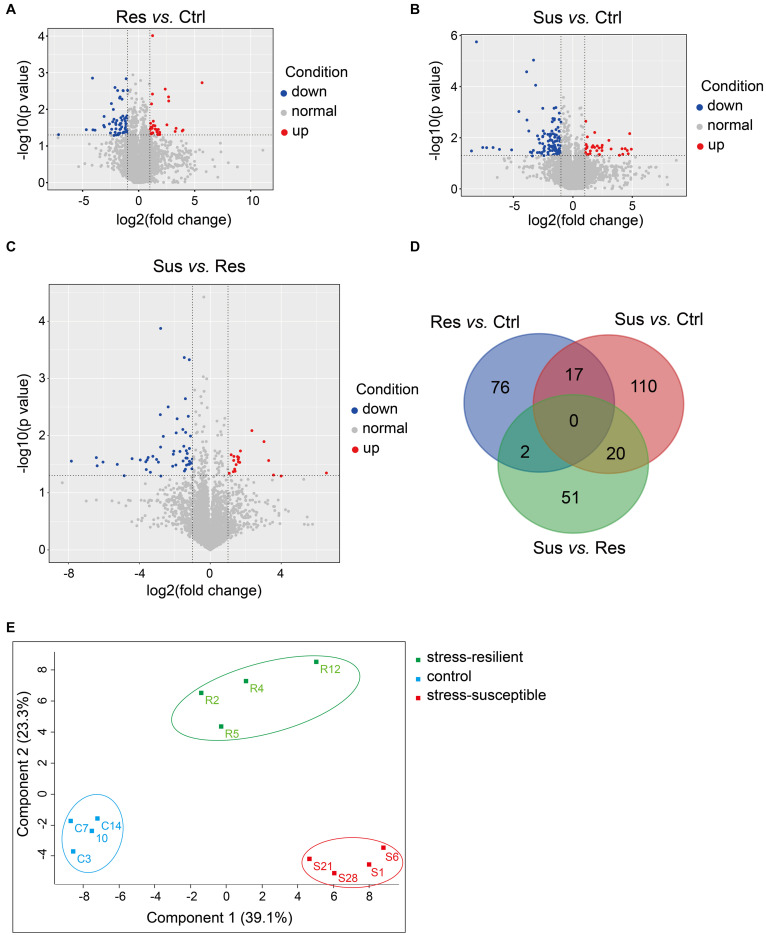
Differential protein analysis from proteomic profiling. **(A–C)** Volcano plot of differentially expressed proteins. Blue points respected the downregulated proteins with no greater than 0.5-fold changes at *P* < 0.05, and red points were considered as the upregulated proteins with at least 2.0-fold changes at *P* < 0.05. **(D)** Venn diagrams revealing the number of altered proteins in the three groups. **(E)** PCA score plot showed obvious differences in the control group, stress-susceptible group, and stress-resilient group. Sus, stress-susceptible; Res, stress-resilient; Ctrl, control.

To show the variation in protein expression between control rats and CUS treatment rats, we further drew the volcano map, as illustrated in Figures 3A–C. Compared with the control group, 35 proteins were upregulated, and 112 proteins were downregulated in the stress-susceptible group, and 33 proteins were upregulated, and 62 proteins were downregulated in the stress-resilient group. Meanwhile, 20 proteins were upregulated, and 53 proteins were downregulated in stress-susceptible versus stress-resilient groups. By further screening the differential proteins among the three groups, we found 17 proteins that showed both different expressions in stress-susceptible versus control groups and stress-resilient versus control groups. Additionally, two proteins were prominently upregulated in the stress-resilient rats compared with the control and stress-susceptible rats. In the stress-susceptible group, three proteins were evidently upregulated, and 17 proteins were downregulated compared with the control group and the stress-resilient group (Table 2 and Figure 3D). The principal component analysis (PCA) was performed to reflect the protein differences among samples and the variation between samples in the group. Protein data were used for PCA analysis. In this case, the intensities of all detected peptides were set as variables, and the three groups were set as observations. PCA score plot showed distinct separation in the three groups (Figure 3E).

**TABLE 2:** List of proteins significantly altered between groups.

Gene symbol	Protein description	Stress vulnerability	Changes
Lias	Lipoyl synthase, mitochondrial precursor	Stress susceptible	↑
Babam1	BRISC and BRCA1-A complex member 1 isoform X1	Stress susceptible	↑
Cnnm2	Metal transporter CNNM2	Stress susceptible	↑
Sqrdl	Sulfide:quinone oxidoreductase, mitochondrial isoform	Stress susceptible	↓
Dcun1d3	DCN1-like protein 3 isoform X1	Stress susceptible	↓
Sidt1	SID1 transmembrane family member 1 isoform X2	Stress susceptible	↓
Cecr5	Cat eye syndrome critical region protein 5	Stress susceptible	↓
Hars2	Probable histidine–tRNA ligase, mitochondrial	Stress susceptible	↓
Prpf3	U4/U6 small nuclear ribonucleoprotein Prp3 isoform X4	Stress susceptible	↓
Maged1	Melanoma-associated antigen D1	Stress susceptible	↓
Vps41	Vacuolar protein sorting-associated protein 41 homolog	Stress susceptible	↓
LOC102556574	Serrate RNA effector molecule homolog	Stress susceptible	↓
Dnajc7	DnaJ homolog subfamily C member 7 isoform X2	Stress susceptible	↓
Atrn	Attractin precursor	Stress susceptible	↓
Tspyl4	Testis-specific Y-encoded-like protein 4	Stress susceptible	↓
Scamp4	Secretory carrier-associated membrane protein 4	Stress susceptible	↓
Camk2n1	Calcium/calmodulin-dependent protein kinase II inhibitor 1	Stress susceptible	↓
Caap1	Caspase activity and apoptosis inhibitor 1 isoform X1	Stress susceptible	↓
Mavs	Mitochondrial antiviral-signaling protein isoform X1	Stress susceptible	↓
Fn1	Fibronectin isoform X10	Stress susceptible	↓
NEWGENE_2116	Acidic leucine-rich nuclear phosphoprotein 32 family member A isoform X4	Stress resilient	↑
Rpl30l1	60S ribosomal protein L30-like	Stress resilient	↑

*↑/↓, significant increase or decrease.*

### Clustering Analysis of Differentially Expressed Proteins

**FIGURE 4:**
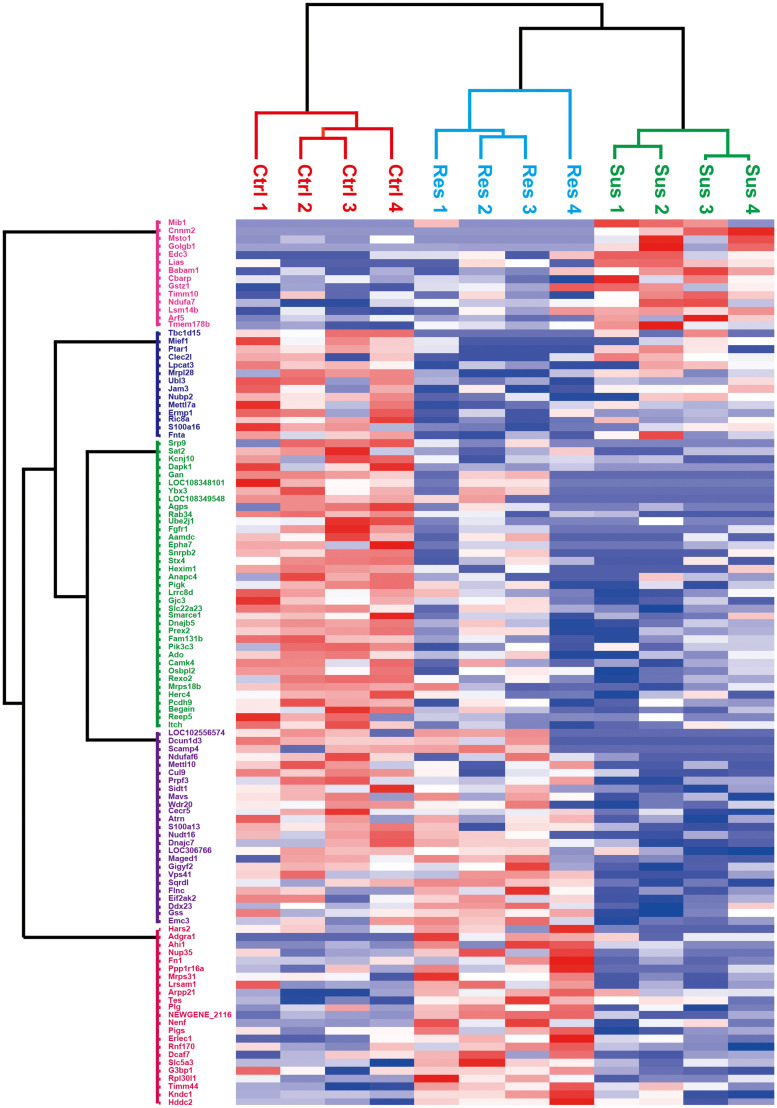
Heatmap of differential proteins in the stress-susceptible group, the stress-resilient group, and the control group. The x-axis was represented for the information of samples, and the y-axis was described for the information of differentially expressed proteins. Higher expressions are indicated by red and lower by blue. The stress-susceptible group, the stress-resilient group, and the control group were clustered into three distinct groups.

We performed hierarchical clustering analysis on the differentially expressed proteins, and the result is displayed in Figure 4. In this study, sample information was on the x-axis, and differential proteins were on the y-axis. The color depth indicated the expression levels. Red was used to indicate prominent upregulated differential proteins, and blue represented apparent downregulated differential proteins. The stress-susceptible group, the stress-resilient group, and the control group were clustered into three distinct groups. These differentially expression proteins were divided into five clusters based on their biological process (BP). The first cluster analysis was mainly related to the BP of tube formation and mitochondrion organization. The second and third cluster analyses were related to phospholipid biosynthetic process, proteolysis, myelination, and axon ensheathment. The fourth cluster was mainly related to the BP of chemokine production and regulation of chemokine production. The fifth cluster was associated with cellular component disassembly. Proteins with similar expression patterns may have similar functions or participate in the same biological pathways.

### Bioinformatics Analysis of Differentially Expressed Proteins

**FIGURE 5:**
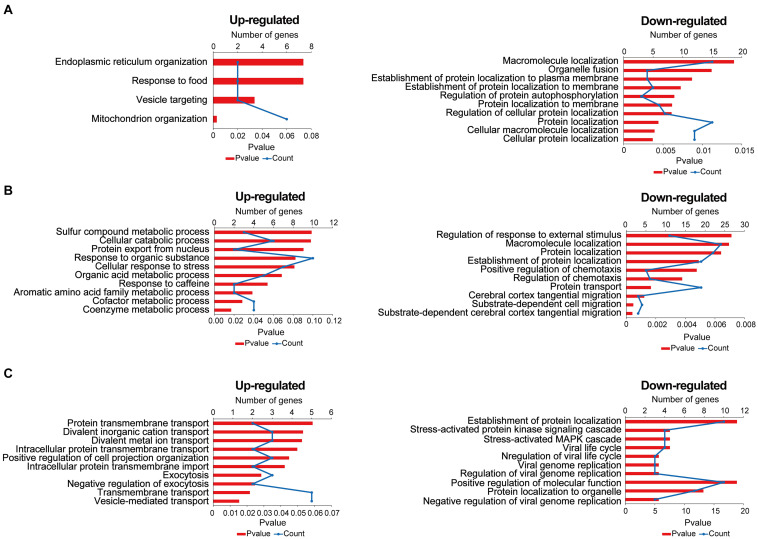
The biological process of differentially expressed proteins in the three comparison groups. **(A)** Stress-resilient versus control. **(B)** Stress-susceptible versus control. **(C)** Stress-susceptible versus stress-resilient. Red represented the *P-*value, and blue represented the number of genes.

Gene Ontology (GO) annotation includes a BP, cell composition, and molecular function. In this research, we mainly focused on BP; the result of GO annotation is shown in Figure 5. In the comparisons of stress-resilient and control groups, upregulated proteins were mainly associated with mitochondrion organization, and downregulated proteins mainly participated in protein localization, macromolecule localization, and so on (Figure 5A). In the comparisons of stress-susceptible and control groups, the BP of upregulated proteins were related to coenzyme metabolic process and cofactor metabolic process. The BP of downregulated proteins mainly participated in the protein transport, substrate-dependent cerebral cortex tangential migration, and so on (Figure 5B). In the comparisons of stress-susceptible and stress-resilient groups, upregulated proteins were related to exocytosis, transmembrane transport, and vesicle-mediated transport protein (Figure 5C).

**FIGURE 6:**
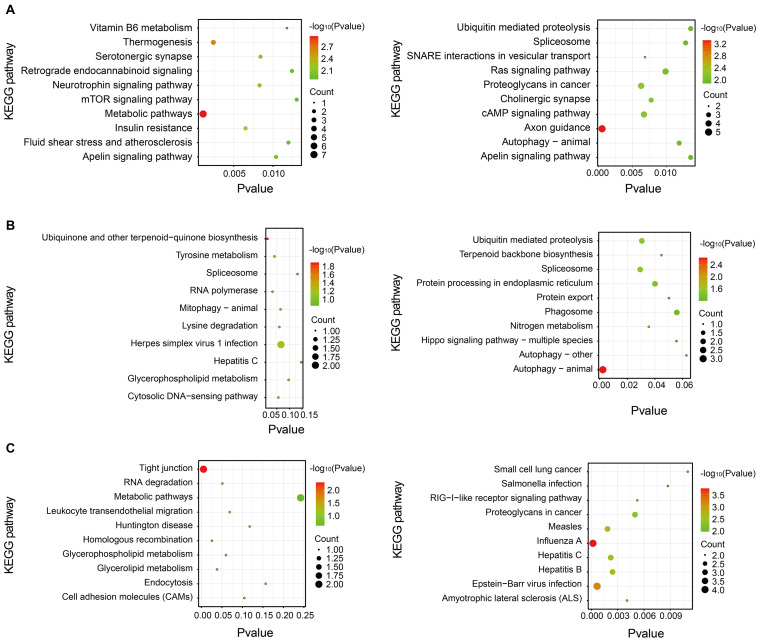
Kyoto Encyclopedia of Genes and Genomes (KEGG) pathway enrichment analysis of differentially expressed proteins. The *P*-value was on the x-axis. The color gradient represented the size of the *P*-value negative log (base 10), and the color gradually changed from green to red. The color was closer to red, the *P-*value was smaller, which corresponded to the KEGG pathway enrichment to a higher degree. **(A)** Stress-resilient versus control. **(B)** Stress-susceptible versus control. **(C)** Stress-susceptible versus stress-resilient. Red represented the *P-*value, and blue represented the number of genes.

Pathway analysis is essential to systematically and comprehensively understand the BP of cells, the occurrence mechanism of diseases, and the action mechanism of the drug. We anotated the pathways of these differentially expressed proteins by the Kyoto Encyclopedia of Genes and Genomes (KEGG). The potential metabolic or signaling pathways that these proteins participated in could be found, which helped to show a series of protein changes from the cell surface to the nucleus, and reveal a series of academic events and action factors involved in this process. The result of KEGG pathway analysis is shown in Figure 6. The KEGG pathway analysis indicated that the upregulated proteins in the stress-resilient group were mainly involved in metabolic pathways. The downregulated proteins were mainly involved in axon guidance, and autophagy—animal (Figure 6A). Compared with control group, the upregulated proteins in stress-susceptible rats were mainly related to the ubiquinone and other terpenoid-quinone biosynthesis, and the downregulated proteins were related to autophagy—animal (Figure 6B). The differentially expressed proteins between stress-resilient and stress-susceptible groups were mainly involved in tight junction and influenza A (Figure 6C).

## Discussion

Exposed to identical stress stimuli, a part of individuals display negative psychology and behavior, whereas some individuals do not appear to have the same symptoms. Given the individual difference in facing stress, we aimed to find stress-susceptible and stress-resilient proteins. In this research, we focused on these proteins that they were obviously upregulated or downregulated in the stress-susceptible group compared with the controls, whereas they were relatively downregulated or upregulated in the stress-resilient group compared with the stress-susceptible group. Through proteomics and statistical analysis, we ultimately obtained 22 stress-related proteins. According to results of clinical trials, these proteins were closely related to schizophrenia, cancer, and other diseases (Rose et al., 2014; Bruun et al., 2018; Zhao et al., 2020). The relationship between these stress-related proteins and depression has not been reported and need to be further explored.

Lias was obviously upregulated in the stress-susceptible group. Lias is involved in the endogenous synthesis of lipoic acid, which is considered as a potent mitochondrial antioxidant (Padmalayam et al., 2009). Researches have suggested that the mitochondria plays a vital role in synaptic plasticity and cellular resilience (Bansal and Kuhad, 2016). Mitochondrial dysfunction is associated with depression. Treatment of the mitochondria decreased the reduction of sucrose consumption and the immobility time in the FST and TST of lipopolysaccharide-exposed mice (Wang et al., 2019). The mutation of Lias leads to an overall disturbance in the antioxidant defense network, insulin resistance, and mitochondrial dysfunction (Burr et al., 2016; Pöntinen et al., 2017). Although a link between Lias and depression has not been directly reported, we inferred that the increased expression levels of Lias may be correlated with the occurrence of depression.

The expression of Cnnm2 was increased in the stress-susceptible group. Cnnm2 is a vital candidate gene related to magnesium ion (Mg^2+^) balance, which is mutated in dominant hypomagnesemia (Stuiver et al., 2011). Clinical data suggested that Mg^2+^ supplementation has been beneficial in preeclampsia, migraine, depression, coronary artery disease, and asthma (de Baaij et al., 2015). Structural magnetic imaging data in patients suggested that Cnnm2 risk variant rs7914558 impacted neural systems associated with social cognition (Rose et al., 2014; de Baaij et al., 2015). Sun et al. (2019) found that dietary magnesium intake was inverse with the risk of depression. We speculate that the upregulation of Cnnm2 may lead to a stress-susceptible phenotype.

According to the relative quantitative results, the Maged1 levels were significantly lower in the stress-susceptible group than in the control group and stress-resilient group. Bioinformatic analysis revealed that Maged1 was involved in the KEGG pathway of organismal systems, nervous system, and neurotrophin signaling pathway. Clinical and basic studies have shown that the expression of neurotrophic factors (NTF) and the activity of receptor will decrease in depression, which can be reversed by antidepressant treatment (Buttenschøn et al., 2015). Brain-derived neurotrophic factor (BDNF) is one of the important NTFs and has gained wide attention. External signals mainly regulated the expression of BDNF through the cAMP/PKA–CREB–BDNF pathway, further regulating the growth and survival of cells, and influenced the occurrence and development of depression. It is also generally reported that depression is associated with abnormalities in 5-HT. 5-HT may regulate the CREB–BDNF pathway through 5-HT1A receptors (Yoo et al., 2017). Maged1 has been reported to play an essential role in the central nervous system in both developmental and adult stages (Mouri et al., 2013; Yang et al., 2015). Limited researches have reported that Maged1 knocked mice-displayed depression-like behaviors by serotonin transporter (SERT) ubiquitylation. Furthermore, the overexpression of Maged1 led to a decrease in serotonin uptake activity and SERT protein level but an increase in ubiquitylated SERT (Mouri et al., 2012; Lu et al., 2020). We infer that Maged1 plays an important role in depression through effecting the 5-HT systems. Maged1 has the potential to be a biomarker for distinguishing stress-susceptible rats and stress-resilient rats.

In organisms, different proteins coordinate with each other to complete a series of biochemical reactions to perform their biological functions. Therefore, pathway analysis is necessary for comprehensively understanding the BPs of cells, disease mechanisms, or drug action mechanisms. The KEGG pathway enrichment analysis of differentially expressed proteins showed that metabolic pathways and axon guidance were closely related to SR, and autophagy was related to stress susceptibility. There is accumulating evidence indicating that dysfunction of mitochondrial energy metabolism is associated with the occurrence and development of depression. The pathogenesis of depression was related to the changes in molecular level of mitochondrial energy metabolism. Mitochondrial DNA defects and low level of adenosine triphosphate (ATP), mitochondrial membrane potential drops, and excess production of reactive oxygen species can directly or indirectly affect mitochondrial energy metabolism resulting in depression (Cao et al., 2013; Tobe, 2013; Vaváková et al., 2015; Tymofiyeva et al., 2018).

Autophagy is the self-clearance of aging, inactivation, and damaged structures within cells, characterized by the formation of autophagosomes. The occurrence of depression is related to neuronal autophagy (Jung et al., 2020). When autophagosomes and microtubule-associated protein 1 light chain 3 (LC3) increased in hippocampal neuron, the number of hippocampus neurons decreased, and apoptosis rate increased. Autophagy shows bidirectional control on depression. At the basic level, autophagy is crucial to stabilize and ensure the normal life activities of cells, which is conducive to the survival of neurons (Shen and Ganetzky, 2009). Once autophagy is overactivated, it will degrade too many organelles or its own proteins, which will exceed its compensatory range resulting in cell death (Shu et al., 2019).

In this study, we explored the potential correlation between differentially expressed proteins and depression by literature. Although the other remaining proteins have not been reported to be associated with depression, they provided the new targets for studying depression. Furthermore, we will verify the protein function in animal and cell levels. Strengthening basic research and clarifying the pathogenesis of depression have a significant role in developing safer and more effective antidepressants. Proteomics analysis of the hippocampus contributes to revealing the pathogenesis of depression at the cellular level, identifying more informative biomarkers, and elucidating the mechanisms of SR and stress susceptible.

## Conclusion

In this study, a label-free quantitative proteomics technique was utilized for the testing and identification of hippocampus proteins. Several proteins were differentially expressing levels in stress-susceptible rats, stress-resilient rats, and control rats. We finally discovered 20 stress-susceptible proteins and two stress-resilient proteins. Further validation and investigation are required to illuminate the results. Bioinformatics analysis revealed that BPs of these differential proteins had different enrichment characteristics, mainly including metabolic pathways, axon guidance, and autophagy. Stress-related proteins contribute to finding biomarkers and revealing potential protein targets of antidepressant drugs.

## Data Availability Statement

The raw data supporting the conclusions of this article will be made available by the authors, without undue reservation.

## Ethics Statement

The animal study was reviewed and approved by Peking Union Medical College and Chinese Academy of Medical Sciences.

## Author Contributions

YaZ contributed to writing the original draft and statistical analysis. XZ, NL, SR, and HW participated in the animal experiments. NZ, CX, and XiY performed the sample preparation. ZZ and YiZ helped to analyze the original data. YaZ and YiZ mainly revised the manuscript. ZW and NC designed the research and were contributors to financial support. All authors contributed to manuscript revision, and read and approved the submitted version.

## Conflict of Interest

The authors declare that the research was conducted in the absence of any commercial or financial relationships that could be construed as a potential conflict of interest.

## Footnotes

1 www.uniprot.org/
