# Supplementary material for: Comparative Proteomic Characterization of Ventral Hippocampus in Susceptible and Resilient Rats Subjected to Chronic Unpredictable Stress
Source: Front Neurosci. 2021 Jun 17;15:675430. doi: 10.3389/fnins.2021.675430 (PMC8249003; doi:10.3389/fnins.2021.675430)
Supplement: Supplementary file 1 [file Table_1.docx]

Table S1. Chronic unpredictable stress procedure

| Day | Stressors |
| --- | --- |
| Day1 | Swim stress 10 ℃, 5min; cage tilt 45° overnight |
| Day2 | Restraint 4 h; reversal of the light/dark cycle (light off 12 h and light on overnight) |
| Day3 | Electric shock, 3 min; shaker stress 2 h |
| Day4 | Tail pinch 1 min; food deprivation 24 h |
| Day5 | Isolation 48 h; water deprivation 24 h |
| Day6 | Ultrasonic stress 2 h; isolation 24 h |
| Day7 | Crowding overnight (8 rats per cage); wet bedding overnight (200 mL water spilled onto 100 g sawdust bedding) |
