# Supplementary material for: Comparative Proteomic Characterization of Ventral Hippocampus in Susceptible and Resilient Rats Subjected to Chronic Unpredictable Stress
Source: Front Neurosci. 2021 Jun 17;15:675430. doi: 10.3389/fnins.2021.675430 (PMC8249003; doi:10.3389/fnins.2021.675430)
Supplement: Supplementary file 2 [file Table_2.docx]

**Table S2. Differentially expressed proteins in stress-susceptible *versus* control**.

| Gene symbol |  | Protein Description | *P*-values | Fold change | Significant |
| --- | --- | --- | --- | --- | --- |
| Adgra1 |  | adhesion G protein-coupled receptor A1 isoform X2 | 0.03 | 23.82 | up-regulated |
| Arpp21 |  | cAMP-regulated phosphoprotein 21 isoform X17 | 0.03 | 4.62 | up-regulated |
| Gstz1 |  | maleylacetoacetate isomerase | 0.01 | 2.33 | up-regulated |
| Usp30 |  | ubiquitin carboxyl-terminal hydrolase 30 isoform X2 | 0.03 | 4.04 | up-regulated |
| Lias |  | lipoyl synthase, mitochondrial precursor | 0.01 | 27.47 | up-regulated |
| Babam1 |  | BRISC and BRCA1-A complex member 1 isoform X1 | 0.01 | 3.57 | up-regulated |
| Cnnm2 |  | metal transporter CNNM2 | 0.01 | 8.17 | up-regulated |
| Tmem178b |  | transmembrane protein 178B precursor | 0.02 | 2.26 | up-regulated |
| Rps6ka2 |  | ribosomal protein S6 kinase alpha-2 | 0.05 | 4.72 | up-regulated |
| Ndufa7 |  | NADH dehydrogenase [ubiquinone] 1 alpha subcomplex subunit 7 | 0.03 | 2.77 | up-regulated |
| Mtfp1 |  | mitochondrial fission process protein 1 | 0.05 | 2.40 | up-regulated |
| Tenm2 |  | teneurin-2 | 0.04 | 2.04 | up-regulated |
| Get4 |  | Golgi to ER traffic protein 4 homolog isoform X1 | 0.05 | 4.31 | up-regulated |
| Trnt1 |  | CCA tRNA nucleotidyltransferase 1, mitochondrial isoform X1 | 0.04 | 2.73 | up-regulated |
| Ttc19 |  | tetratricopeptide repeat protein 19, mitochondrial | 0.04 | 25.20 | up-regulated |
| LOC299282 |  | Serine protease inhibitor | 0.02 | 5.54 | up-regulated |
| RGD1566085 |  | pyridoxal kinase-like isoform X2 | 0.03 | 21.10 | up-regulated |
| Arf2 |  | ADP-ribosylation factor 2 | 0.03 | 30.16 | up-regulated |
| Lsm14b |  | protein LSM14 homolog B isoform X3 | 0.00 | 2.13 | up-regulated |
| Edc3 |  | enhancer of mRNA-decapping protein 3 | 0.02 | 4.15 | up-regulated |
| Mtss1l |  | MTSS1-like protein | 0.02 | 2.17 | up-regulated |
| Gng3 |  | guanine nucleotide-binding protein G(I)/G(S)/G(O) subunit gamma-3 | 0.03 | 5.47 | up-regulated |
| Atpaf2 |  | ATP synthase mitochondrial F1 complex assembly factor 2 | 0.04 | 2.10 | up-regulated |
| RGD1309534 |  | ester hydrolase C11orf54 homolog | 0.05 | 4.51 | up-regulated |
| Adar |  | double-stranded RNA-specific adenosine deaminase isoform X4 | 0.04 | 18.31 | up-regulated |
| Msi1 |  | RNA-binding protein Musashi homolog 1 | 0.05 | 15.06 | up-regulated |
| Gstm2 |  | glutathione S-transferase Mu 2 | 0.02 | 3.06 | up-regulated |
| Tph2 |  | tryptophan 5-hydroxylase 2 | 0.05 | 4.47 | up-regulated |
| LOC679539 |  | ubiquitin-conjugating enzyme E2 variant 1 | 0.02 | 3.55 | up-regulated |
| Prkaa2 |  | 5'-AMP-activated protein kinase catalytic subunit alpha-2 | 0.02 | 5.41 | up-regulated |
| Gpsm1 |  | G-protein-signaling modulator 1 isoform a | 0.02 | 2.29 | up-regulated |
| Dnajc27 |  | dnaJ homolog subfamily C member 27 | 0.03 | 17.54 | up-regulated |
| Mib1 |  | E3 ubiquitin-protein ligase MIB1 | 0.03 | 9.28 | up-regulated |
| Acot2 |  | acyl-coenzyme A thioesterase 2, mitochondrial | 0.03 | 2.12 | up-regulated |
| Gab1 |  | GRB2-associated-binding protein 1 | 0.02 | 3.23 | up-regulated |
| Pycrl |  | pyrroline-5-carboxylate reductase 3 | 0.01 | 1.65 | up-regulated |
| Plcl2 |  | inactive phospholipase C-like protein 2 | 0.04 | 1.74 | up-regulated |
| Cox5b |  | cytochrome c oxidase subunit 5B, mitochondrial precursor | 0.04 | 1.69 | up-regulated |
| Kpna1 |  | importin subunit alpha-5 isoform X2 | 0.00 | 1.67 | up-regulated |
| RGD1311739 |  | UPF0687 protein C20orf27 homolog isoform X1 | 0.01 | 1.77 | up-regulated |
| Cnpy2 |  | protein canopy homolog 2 precursor | 0.05 | 1.76 | up-regulated |
| Pde1a |  | calcium/calmodulin-dependent 3',5'-cyclic nucleotide phosphodiesterase 1A | 0.04 | 1.93 | up-regulated |
| Ahcy |  | adenosylhomocysteinase | 0.00 | 1.60 | up-regulated |
| Nop56 |  | nucleolar protein 56 | 0.03 | 1.84 | up-regulated |
| Ankfy1 |  | rabankyrin-5 | 0.00 | 1.80 | up-regulated |
| Pnmal2 |  | PNMA-like protein 2 | 0.04 | 1.56 | up-regulated |
| Pde6d |  | retinal rod rhodopsin-sensitive cGMP 3',5'-cyclic phosphodiesterase subunit delta isoform X1 | 0.00 | 1.62 | up-regulated |
| LOC100911365 |  | Parkinson disease 7 domain-containing protein 1 isoform X2 | 0.03 | 1.56 | up-regulated |
| Fam131b |  | protein FAM131B | 0.01 | 0.38 | down-regulated |
| Rab34 |  | ras-related protein Rab-34 | 0.00 | 0.11 | down-regulated |
| Hexim1 |  | protein HEXIM1 | 0.01 | 0.23 | down-regulated |
| Stx4 |  | syntaxin-4 | 0.01 | 0.17 | down-regulated |
| Cul9 |  | cullin-9 isoform X4 | 0.00 | 0.17 | down-regulated |
| Fgfr1 |  | fibroblast growth factor receptor 1 isoform X6 | 0.02 | 0.15 | down-regulated |
| Dnajb5 |  | dnaJ homolog subfamily B member 5 isoform X2 | 0.00 | 0.33 | down-regulated |
| Ube2j1 |  | ubiquitin-conjugating enzyme E2 J1 isoform X1 | 0.04 | 0.14 | down-regulated |
| Pcdh9 |  | protocadherin-9 isoform X4 | 0.01 | 0.45 | down-regulated |
| Dapk1 |  | death-associated protein kinase 1 isoform X2 | 0.04 | 0.16 | down-regulated |
| Slc35b1 |  | solute carrier family 35 member B1 | 0.03 | 0.38 | down-regulated |
| Snrpb2 |  | U2 small nuclear ribonucleoprotein B'' isoform X1 | 0.01 | 0.18 | down-regulated |
| Epha7 |  | ephrin type-A receptor 7 isoform X1 | 0.03 | 0.18 | down-regulated |
| Sqrdl |  | sulfide:quinone oxidoreductase, mitochondrial isoform X1 | 0.01 | 0.19 | down-regulated |
| Dcun1d3 |  | DCN1-like protein 3 isoform X1 | 0.00 | 0.07 | down-regulated |
| Sidt1 |  | SID1 transmembrane family member 1 isoform X2 | 0.01 | 0.26 | down-regulated |
| Fn1 |  | fibronectin isoform X10 | 0.01 | 0.26 | down-regulated |
| Cecr5 |  | cat eye syndrome critical region protein 5 | 0.02 | 0.31 | down-regulated |
| Hars2 |  | probable histidine--tRNA ligase, mitochondrial | 0.01 | 0.08 | down-regulated |
| Prpf3 |  | U4/U6 small nuclear ribonucleoprotein Prp3 isoform X4 | 0.02 | 0.26 | down-regulated |
| Maged1 |  | melanoma-associated antigen D1 | 0.04 | 0.46 | down-regulated |
| Vps41 |  | vacuolar protein sorting-associated protein 41 homolog | 0.02 | 0.17 | down-regulated |
| LOC102556574 |  | serrate RNA effector molecule homolog | 0.03 | 0.01 | down-regulated |
| Dnajc7 |  | dnaJ homolog subfamily C member 7 isoform X2 | 0.03 | 0.36 | down-regulated |
| Atrn |  | attractin precursor | 0.04 | 0.43 | down-regulated |
| Tspyl4 |  | testis-specific Y-encoded-like protein 4 | 0.03 | 0.10 | down-regulated |
| Scamp4 |  | secretory carrier-associated membrane protein 4 isoform X1 | 0.02 | 0.01 | down-regulated |
| Camk2n1 |  | calcium/calmodulin-dependent protein kinase II inhibitor 1 | 0.02 | 0.01 | down-regulated |
| Mavs |  | mitochondrial antiviral-signaling protein isoform X1 | 0.02 | 0.30 | down-regulated |
| Herc4 |  | probable E3 ubiquitin-protein ligase HERC4 isoform X3 | 0.03 | 0.43 | down-regulated |
| Lrrc8d |  | volume-regulated anion channel subunit LRRC8D isoform X1 | 0.01 | 0.35 | down-regulated |
| Rexo2 |  | oligoribonuclease, mitochondrial precursor | 0.01 | 0.41 | down-regulated |
| Slc22a23 |  | solute carrier family 22 member 23 | 0.00 | 0.34 | down-regulated |
| NEWGENE_1308105 |  | kinase suppressor of Ras 1 isoform X7 | 0.01 | 0.49 | down-regulated |
| Osbpl2 |  | oxysterol-binding protein-related protein 2 isoform X2 | 0.01 | 0.45 | down-regulated |
| Gan |  | gigaxonin isoform X1 | 0.00 | 0.10 | down-regulated |
| Prex2 |  | phosphatidylinositol 3,4,5-trisphosphate-dependent Rac exchanger 2 protein isoform X1 | 0.00 | 0.34 | down-regulated |
| Camk4 |  | calcium/calmodulin-dependent protein kinase type IV isoform X3 | 0.01 | 0.46 | down-regulated |
| Ermp1 |  | endoplasmic reticulum metallopeptidase 1 | 0.03 | 0.45 | down-regulated |
| Ado |  | 2-aminoethanethiol dioxygenase | 0.01 | 0.40 | down-regulated |
| Mrps18b |  | 28S ribosomal protein S18b, mitochondrial | 0.00 | 0.45 | down-regulated |
| Rras |  | ras-related protein R-Ras | 0.05 | 0.31 | down-regulated |
| Kcnj10 |  | ATP-sensitive inward rectifier potassium channel 10 isoform X1 | 0.02 | 0.35 | down-regulated |
| Sat2 |  | diamine acetyltransferase 2 isoform X2 | 0.02 | 0.19 | down-regulated |
| Gjc3 |  | gap junction gamma-3 protein isoform X1 | 0.01 | 0.36 | down-regulated |
| LOC108349548 |  | guanine nucleotide-binding protein G(I)/G(S)/G(O) subunit gamma-5 | 0.00 | 0.00 | down-regulated |
| Begain |  | brain-enriched guanylate kinase-associated protein isoform X7 | 0.01 | 0.47 | down-regulated |
| Robo1 |  | roundabout homolog 1 isoform X11 | 0.03 | 0.34 | down-regulated |
| LOC108348101 |  | chloride channel protein 2 isoform X2 | 0.01 | 0.14 | down-regulated |
| Pigk |  | GPI-anchor transamidase isoform X1 | 0.01 | 0.27 | down-regulated |
| Cpeb4 |  | cytoplasmic polyadenylation element-binding protein 4 isoform X3 | 0.02 | 0.36 | down-regulated |
| Aamdc |  | mth938 domain-containing protein isoform X1 | 0.01 | 0.17 | down-regulated |
| LOC306766 |  | putative monooxygenase p33MONOX | 0.04 | 0.40 | down-regulated |
| Git2 |  | ARF GTPase-activating protein GIT2 isoform X11 | 0.00 | 0.45 | down-regulated |
| Srp9 |  | signal recognition particle 9 kDa protein | 0.02 | 0.01 | down-regulated |
| Aamp |  | angio-associated migratory cell protein isoform X3 | 0.05 | 0.33 | down-regulated |
| Ybx3 |  | Y-box-binding protein 3 | 0.00 | 0.04 | down-regulated |
| Agps |  | alkyldihydroxyacetonephosphate synthase, peroxisomal | 0.04 | 0.08 | down-regulated |
| Dpp8 |  | dipeptidyl peptidase 8 isoform X2 | 0.05 | 0.45 | down-regulated |
| S100a13 |  | protein S100-A13 | 0.00 | 0.37 | down-regulated |
| Wdr91 |  | WD repeat-containing protein 91 | 0.04 | 0.44 | down-regulated |
| Ccsap |  | centriole, cilia and spindle-associated protein isoform X1 | 0.04 | 0.48 | down-regulated |
| Faf1 |  | FAS-associated factor 1 | 0.00 | 0.31 | down-regulated |
| Ndufaf6 |  | NADH dehydrogenase (ubiquinone) complex I, assembly factor 6 precursor | 0.00 | 0.07 | down-regulated |
| Abcb9 |  | ATP-binding cassette sub-family B member 9 | 0.01 | 0.27 | down-regulated |
| Tkfc |  | triokinase/FMN cyclase isoform X2 | 0.05 | 0.50 | down-regulated |
| Fam91a1 |  | protein FAM91A1 | 0.03 | 0.46 | down-regulated |
| Pde4a |  | cAMP-specific 3',5'-cyclic phosphodiesterase 4A | 0.03 | 0.39 | down-regulated |
| Ube3c |  | ubiquitin-protein ligase E3C isoform X1 | 0.03 | 0.45 | down-regulated |
| Nrp1 |  | neuropilin-1 isoform X3 | 0.04 | 0.39 | down-regulated |
| Zfp512 |  | zinc finger protein 512 isoform X2 | 0.01 | 0.28 | down-regulated |
| Ppp6r1 |  | serine/threonine-protein phosphatase 6 regulatory subunit 1 | 0.03 | 0.36 | down-regulated |
| Irgm |  | immunity-related GTPase family M protein isoform X2 | 0.04 | 0.40 | down-regulated |
| Nudt16 |  | U8 snoRNA-decapping enzyme | 0.00 | 0.37 | down-regulated |
| Hectd1 |  | E3 ubiquitin-protein ligase HECTD1 isoform X5 | 0.04 | 0.37 | down-regulated |
| Mettl10 |  | protein-lysine N-methyltransferase METTL10 isoform X4 | 0.01 | 0.13 | down-regulated |
| Samsn1 |  | SAM domain-containing protein SAMSN-1 isoform X4 | 0.03 | 0.03 | down-regulated |
| Adi1 |  | 1,2-dihydroxy-3-keto-5-methylthiopentene dioxygenase | 0.02 | 0.43 | down-regulated |
| Scaf8 |  | protein SCAF8 | 0.03 | 0.24 | down-regulated |
| Atg13 |  | autophagy-related protein 13 | 0.04 | 0.09 | down-regulated |
| Adam10 |  | disintegrin and metalloproteinase domain-containing protein 10 isoform X2 | 0.00 | 0.42 | down-regulated |
| Wdr20 |  | WD repeat-containing protein 20 isoform X7 | 0.01 | 0.32 | down-regulated |
| Ccdc92 |  | coiled-coil domain-containing protein 92 isoform X1 | 0.04 | 0.38 | down-regulated |
| Klhl22 |  | kelch-like protein 22 isoform X3 | 0.01 | 0.30 | down-regulated |
| Sf3a2 |  | splicing factor 3A subunit 2 isoform X1 | 0.02 | 0.32 | down-regulated |
| Bsdc1 |  | BSD domain-containing protein 1 | 0.04 | 0.39 | down-regulated |
| Emc7 |  | ER membrane protein complex subunit 7 isoform X1 | 0.02 | 0.29 | down-regulated |
| Snx19 |  | sorting nexin-19 | 0.03 | 0.27 | down-regulated |
| Epha5 |  | ephrin type-A receptor 5 isoform X5 | 0.02 | 0.34 | down-regulated |
| Igsf11 |  | immunoglobulin superfamily member 11 precursor | 0.04 | 0.24 | down-regulated |
| Mrps5 |  | 28S ribosomal protein S5, mitochondrial | 0.01 | 0.27 | down-regulated |
| LOC108348111 |  | succinate dehydrogenase assembly factor 1, mitochondrial | 0.01 | 0.24 | down-regulated |
| Pdcd4 |  | programmed cell death protein 4 isoform X1 | 0.02 | 0.25 | down-regulated |
| Ccdc43 |  | coiled-coil domain-containing protein 43 | 0.04 | 0.49 | down-regulated |
| Gpr155 |  | integral membrane protein GPR155 isoform X3 | 0.03 | 0.18 | down-regulated |
| Gigyf2 |  | PERQ amino acid-rich with GYF domain-containing protein 2 isoform X3 | 0.00 | 0.50 | down-regulated |
| Fam103a1 |  | RNMT-activating mini protein | 0.04 | 0.13 | down-regulated |
| Vezt |  | vezatin isoform X5 | 0.04 | 0.16 | down-regulated |
| Gspt2 |  | eukaryotic peptide chain release factor GTP-binding subunit ERF3B | 0.02 | 0.49 | down-regulated |
| Nr2c2ap |  | nuclear receptor 2C2-associated protein isoform X1 | 0.04 | 0.23 | down-regulated |
| LOC103691813 |  | acanthoscurrin-1 isoform X1 | 0.03 | 0.00 | down-regulated |
| Zranb2 |  | zinc finger Ran-binding domain-containing protein 2 isoform X2 | 0.01 | 0.29 | down-regulated |
| Scamp2 |  | secretory carrier-associated membrane protein 2 | 0.03 | 0.19 | down-regulated |
| Mrpl46 |  | 39S ribosomal protein L46, mitochondrial | 0.01 | 0.36 | down-regulated |
| Cacna1c |  | voltage-dependent L-type calcium channel subunit alpha-1C isoform X4 | 0.03 | 0.30 | down-regulated |
| Ehd4 |  | EH domain-containing protein 4 | 0.00 | 0.30 | down-regulated |
| Gde1 |  | glycerophosphodiester phosphodiesterase 1 | 0.02 | 0.33 | down-regulated |
| Iscu |  | iron-sulfur cluster assembly enzyme ISCU, mitochondrial | 0.03 | 0.32 | down-regulated |
| Gosr1 |  | Golgi SNAP receptor complex member 1 isoform X1 | 0.02 | 0.27 | down-regulated |
| Pigs |  | GPI transamidase component PIG-S | 0.04 | 0.55 | down-regulated |
| Emc3 |  | ER membrane protein complex subunit 3 | 0.05 | 0.51 | down-regulated |
| Itch |  | E3 ubiquitin-protein ligase Itchy homolog isoform X1 | 0.02 | 0.64 | down-regulated |
| Phgdh |  | D-3-phosphoglycerate dehydrogenase isoform X1 | 0.01 | 0.58 | down-regulated |
| Ppp3r1 |  | calcineurin subunit B type 1 | 0.00 | 0.62 | down-regulated |
| Rida |  | 2-iminobutanoate/2-iminopropanoate deaminase | 0.00 | 0.61 | down-regulated |
| Agap3 |  | arf-GAP with GTPase, ANK repeat and PH domain-containing protein 3 isoform X5 | 0.01 | 0.65 | down-regulated |
| Sfr1 |  | swi5-dependent recombination DNA repair protein 1 homolog | 0.00 | 0.60 | down-regulated |
| Rgs10 |  | regulator of G-protein signaling 10 isoform X1 | 0.03 | 0.60 | down-regulated |
| Mrps30 |  | 28S ribosomal protein S30, mitochondrial | 0.00 | 0.62 | down-regulated |
| Clu |  | clusterin isoform X1 | 0.01 | 0.58 | down-regulated |
| Smpd3 |  | sphingomyelin phosphodiesterase 3 isoform X1 | 0.03 | 0.64 | down-regulated |
| Cacna1a |  | voltage-dependent P/Q-type calcium channel subunit alpha-1A isoform X14 | 0.00 | 0.54 | down-regulated |
| Csnk2b |  | casein kinase II subunit beta | 0.01 | 0.67 | down-regulated |
| Coro7 |  | coronin-7 | 0.04 | 0.57 | down-regulated |
| Ube2h |  | ubiquitin-conjugating enzyme E2 H | 0.03 | 0.59 | down-regulated |
| Ryr2 |  | ryanodine receptor 2 isoform 1 | 0.03 | 0.61 | down-regulated |
| Slc8a2 |  | sodium/calcium exchanger 2 precursor | 0.02 | 0.64 | down-regulated |
| Shisa6 |  | protein shisa-6 homolog isoform X1 | 0.05 | 0.56 | down-regulated |
| LOC103693015 |  | vitamin K epoxide reductase complex subunit 1-like protein 1 isoform X1 | 0.01 | 0.51 | down-regulated |
| Rftn2 |  | raftlin-2 | 0.00 | 0.57 | down-regulated |
| Eftud2 |  | 116 kDa U5 small nuclear ribonucleoprotein component isoform X2 | 0.04 | 0.63 | down-regulated |
| Tm9sf2 |  | transmembrane 9 superfamily member 2 precursor | 0.04 | 0.62 | down-regulated |
| Nrdc |  | nardilysin precursor | 0.01 | 0.62 | down-regulated |
| Ccdc25 |  | coiled-coil domain-containing protein 25 | 0.01 | 0.53 | down-regulated |
| Preb |  | prolactin regulatory element-binding protein | 0.01 | 0.64 | down-regulated |
| Acap2 |  | arf-GAP with coiled-coil, ANK repeat and PH domain-containing protein 2 isoform X4 | 0.02 | 0.57 | down-regulated |
| Itpr1 |  | inositol 1,4,5-trisphosphate receptor type 1 isoform X12 | 0.01 | 0.67 | down-regulated |
| Snap47 |  | synaptosomal-associated protein 47 isoform X1 | 0.05 | 0.61 | down-regulated |
| Tmem41b |  | transmembrane protein 41B isoform X1 | 0.01 | 0.63 | down-regulated |
| Iqgap2 |  | ras GTPase-activating-like protein IQGAP2 isoform X1 | 0.03 | 0.58 | down-regulated |
| Eps15 |  | epidermal growth factor receptor substrate 15 | 0.03 | 0.61 | down-regulated |
| Idi1 |  | isopentenyl-diphosphate Delta-isomerase 1 | 0.01 | 0.52 | down-regulated |
| Pa2g4 |  | proliferation-associated protein 2G4 | 0.01 | 0.52 | down-regulated |
| Prrc1 |  | protein PRRC1 isoform X1 | 0.04 | 0.62 | down-regulated |
| Kalrn |  | kalirin | 0.05 | 0.63 | down-regulated |
| Erc1 |  | ELKS/Rab6-interacting/CAST family member 1 | 0.03 | 0.66 | down-regulated |
| Robo2 |  | roundabout homolog 2 isoform X6 | 0.01 | 0.55 | down-regulated |
| Ica1 |  | islet cell autoantigen 1 isoform X2 | 0.04 | 0.54 | down-regulated |
| Hcfc1 |  | host cell factor 1 | 0.00 | 0.58 | down-regulated |
| Lrrc4b |  | leucine-rich repeat-containing protein 4B isoform X1 | 0.03 | 0.63 | down-regulated |
| Fer |  | tyrosine-protein kinase Fer isoform X2 | 0.03 | 0.62 | down-regulated |
| Fkbp5 |  | peptidyl-prolyl cis-trans isomerase FKBP5 isoform X2 | 0.04 | 0.57 | down-regulated |
| Kcnc1 |  | potassium voltage-gated channel subfamily C member 1 | 0.00 | 0.57 | down-regulated |
| Nav1 |  | neuron navigator 1 isoform X5 | 0.01 | 0.66 | down-regulated |
| Ampd3 |  | AMP deaminase 3 isoform X3 | 0.05 | 0.60 | down-regulated |
| Cmbl |  | carboxymethylenebutenolidase homolog isoform X1 | 0.00 | 0.61 | down-regulated |
| Eif3k |  | eukaryotic translation initiation factor 3 subunit K isoform X1 | 0.01 | 0.60 | down-regulated |
| H2afz |  | histone H2A.Z | 0.03 | 0.59 | down-regulated |
| Gga3 |  | ADP-ribosylation factor-binding protein GGA3 isoform X1 | 0.04 | 0.62 | down-regulated |
| Usp4 |  | ubiquitin carboxyl-terminal hydrolase 4 isoform b | 0.02 | 0.55 | down-regulated |
| Lpcat4 |  | lysophospholipid acyltransferase LPCAT4 | 0.04 | 0.57 | down-regulated |
| Fn3krp |  | ketosamine-3-kinase | 0.02 | 0.64 | down-regulated |
