# Supplementary material for: Comparative Proteomic Characterization of Ventral Hippocampus in Susceptible and Resilient Rats Subjected to Chronic Unpredictable Stress
Source: Front Neurosci. 2021 Jun 17;15:675430. doi: 10.3389/fnins.2021.675430 (PMC8249003; doi:10.3389/fnins.2021.675430)
Supplement: Supplementary file 3 [file Table_3.docx]

**Table S3. Differentially expressed proteins in stress-resilient *versus* control**.

| Gene symbol |  | Protein Description | *P*-values | Fold change | Significant |
| --- | --- | --- | --- | --- | --- |
| NEWGENE_2116 |  | acidic leucine-rich nuclear phosphoprotein 32 family member A isoform X4 | 0.00 | 2.37 | up-regulated |
| Rpl30l1 |  | 60S ribosomal protein L30-like | 0.04 | 2.07 | up-regulated |
| Adgra1 |  | adhesion G protein-coupled receptor A1 isoform X2 | 0.00 | 50.51 | up-regulated |
| Arpp21 |  | cAMP-regulated phosphoprotein 21 isoform X17 | 0.00 | 6.36 | up-regulated |
| Gstz1 |  | maleylacetoacetate isomerase | 0.04 | 2.02 | up-regulated |
| Usp30 |  | ubiquitin carboxyl-terminal hydrolase 30 isoform X2 | 0.05 | 3.71 | up-regulated |
| Psd2 |  | PH and SEC7 domain-containing protein 2 | 0.00 | 5.17 | up-regulated |
| Timm44 |  | mitochondrial import inner membrane translocase subunit TIM44 | 0.00 | 2.35 | up-regulated |
| Tes |  | testin | 0.01 | 6.42 | up-regulated |
| Col4a3bp |  | collagen type IV alpha-3-binding protein | 0.01 | 2.22 | up-regulated |
| Ubap2 |  | ubiquitin-associated protein 2 | 0.02 | 2.48 | up-regulated |
| Cc2d1a |  | coiled-coil and C2 domain-containing protein 1A isoform X3 | 0.02 | 2.09 | up-regulated |
| Trmu |  | mitochondrial tRNA-specific 2-thiouridylase 1 | 0.03 | 5.74 | up-regulated |
| Nenf |  | neudesin precursor | 0.03 | 2.73 | up-regulated |
| Slc5a3 |  | sodium/myo-inositol cotransporter | 0.03 | 2.49 | up-regulated |
| Hddc2 |  | HD domain-containing protein 2 isoform X1 | 0.03 | 2.26 | up-regulated |
| Lpgat1 |  | acyl-CoA:lysophosphatidylglycerol acyltransferase 1 | 0.03 | 2.15 | up-regulated |
| Scrib |  | protein scribble homolog isoform X9 | 0.04 | 2.40 | up-regulated |
| Erlec1 |  | endoplasmic reticulum lectin 1 isoform X1 | 0.03 | 2.32 | up-regulated |
| Coq3 |  | ubiquinone biosynthesis O-methyltransferase, mitochondrial | 0.03 | 2.09 | up-regulated |
| Itih3 |  | inter-alpha-trypsin inhibitor heavy chain H3 isoform X5 | 0.03 | 2.81 | up-regulated |
| Ahi1 |  | jouberin | 0.03 | 10.12 | up-regulated |
| Polr1c |  | DNA-directed RNA polymerases I and III subunit RPAC1 | 0.04 | 15.60 | up-regulated |
| Hax1 |  | HCLS1-associated protein X-1 | 0.04 | 3.38 | up-regulated |
| Wdr45b |  | WD repeat domain phosphoinositide-interacting protein 3 isoform X1 | 0.04 | 2.97 | up-regulated |
| Slc25a18 |  | mitochondrial glutamate carrier 2 | 0.04 | 14.81 | up-regulated |
| Srsf3 |  | serine/arginine-rich splicing factor 3 | 0.04 | 3.10 | up-regulated |
| Tmlhe |  | trimethyllysine dioxygenase, mitochondrial isoform X1 | 0.04 | 9.58 | up-regulated |
| Shtn1 |  | shootin-1 isoform 1 | 0.04 | 3.67 | up-regulated |
| Scyl1 |  | N-terminal kinase-like protein isoform X1 | 0.05 | 2.40 | up-regulated |
| Drg1 |  | developmentally-regulated GTP-binding protein 1 | 0.05 | 2.08 | up-regulated |
| MGC94199 |  | protein C8orf37 homolog isoform X5 | 0.05 | 3.24 | up-regulated |
| Rnasel |  | 2-5A-dependent ribonuclease isoform X1 | 0.05 | 3.28 | up-regulated |
| Kndc1 |  | protein very KIND isoform X2 | 0.01 | 1.60 | up-regulated |
| Tmem178b |  | transmembrane protein 178B precursor | 0.01 | 1.66 | up-regulated |
| Gsta4 |  | glutathione S-transferase alpha-4 | 0.00 | 1.94 | up-regulated |
| D2hgdh |  | D-2-hydroxyglutarate dehydrogenase, mitochondrial | 0.01 | 1.51 | up-regulated |
| Plcd1 |  | 1-phosphatidylinositol 4,5-bisphosphate phosphodiesterase delta-1 isoform X1 | 0.01 | 1.66 | up-regulated |
| Psme1 |  | proteasome activator complex subunit 1 | 0.01 | 1.53 | up-regulated |
| Map2k2 |  | dual specificity mitogen-activated protein kinase kinase 2 | 0.03 | 1.56 | up-regulated |
| Rpl35 |  | 60S ribosomal protein L35 | 0.03 | 1.56 | up-regulated |
| Tsn |  | translin | 0.03 | 1.56 | up-regulated |
| Ppa2 |  | inorganic pyrophosphatase 2, mitochondrial | 0.04 | 1.67 | up-regulated |
| Sdcbp |  | syntenin-1 | 0.04 | 1.56 | up-regulated |
| Scg2 |  | secretogranin-2 isoform X1 | 0.04 | 1.73 | up-regulated |
| Ecsit |  | evolutionarily conserved signaling intermediate in Toll pathway, mitochondrial isoform X4 | 0.04 | 1.91 | up-regulated |
| Cadm3 |  | cell adhesion molecule 3 precursor | 0.04 | 1.70 | up-regulated |
| Ncam2 |  | neural cell adhesion molecule 2 precursor | 0.04 | 1.76 | up-regulated |
| Rps5 |  | 40S ribosomal protein S5 isoform 2 | 0.04 | 1.56 | up-regulated |
| Tm9sf3 |  | transmembrane 9 superfamily member 3 isoform X2 | 0.05 | 1.80 | up-regulated |
| Pycrl |  | pyrroline-5-carboxylate reductase 3 | 0.05 | 1.52 | up-regulated |
| LOC103694855 |  | hemoglobin subunit beta-2-like isoform X1 | 0.05 | 1.60 | up-regulated |
| Plcl2 |  | inactive phospholipase C-like protein 2 | 0.05 | 1.80 | up-regulated |
| Apc |  | adenomatous polyposis coli protein isoform X8 | 0.05 | 1.57 | up-regulated |
| Vps33a |  | vacuolar protein sorting-associated protein 33A | 0.05 | 1.86 | up-regulated |
| Grip1 |  | glutamate receptor-interacting protein 1 | 0.05 | 1.54 | up-regulated |
| Fam131b |  | protein FAM131B | 0.00 | 0.39 | down-regulated |
| Rab34 |  | ras-related protein Rab-34 | 0.00 | 0.38 | down-regulated |
| Hexim1 |  | protein HEXIM1 | 0.00 | 0.31 | down-regulated |
| Stx4 |  | syntaxin-4 | 0.02 | 0.33 | down-regulated |
| Cul9 |  | cullin-9 isoform X4 | 0.02 | 0.49 | down-regulated |
| Fgfr1 |  | fibroblast growth factor receptor 1 isoform X6 | 0.02 | 0.20 | down-regulated |
| Dnajb5 |  | dnaJ homolog subfamily B member 5 isoform X2 | 0.02 | 0.44 | down-regulated |
| Ube2j1 |  | ubiquitin-conjugating enzyme E2 J1 isoform X1 | 0.03 | 0.12 | down-regulated |
| Pcdh9 |  | protocadherin-9 isoform X4 | 0.04 | 0.44 | down-regulated |
| Slc35b1 |  | solute carrier family 35 member B1 | 0.04 | 0.40 | down-regulated |
| Snrpb2 |  | U2 small nuclear ribonucleoprotein B'' isoform X1 | 0.04 | 0.38 | down-regulated |
| Tbc1d15 |  | TBC1 domain family member 15 isoform X2 | 0.00 | 0.06 | down-regulated |
| Ric8a |  | synembryn-A | 0.00 | 0.46 | down-regulated |
| Fnta |  | protein farnesyltransferase/geranylgeranyltransferase type-1 subunit alpha | 0.00 | 0.50 | down-regulated |
| Ptar1 |  | protein prenyltransferase alpha subunit repeat-containing protein 1 | 0.00 | 0.23 | down-regulated |
| Lpcat3 |  | lysophospholipid acyltransferase 5 isoform X1 | 0.00 | 0.27 | down-regulated |
| Nubp2 |  | cytosolic Fe-S cluster assembly factor NUBP2 | 0.00 | 0.32 | down-regulated |
| Ddx19b |  | ATP-dependent RNA helicase DDX19B | 0.01 | 0.36 | down-regulated |
| Sec61a2 |  | protein transport protein Sec61 subunit alpha | 0.01 | 0.19 | down-regulated |
| Mief1 |  | mitochondrial dynamics protein MID51 isoform X1 | 0.01 | 0.21 | down-regulated |
| Car7 |  | carbonic anhydrase 7 isoform X2 | 0.02 | 0.12 | down-regulated |
| Slc7a8 |  | large neutral amino acids transporter small subunit 2 | 0.02 | 0.38 | down-regulated |
| Mtmr12 |  | myotubularin-related protein 12 isoform X1 | 0.02 | 0.46 | down-regulated |
| Cacnb2 |  | voltage-dependent L-type calcium channel subunit beta-2 isoform X11 | 0.02 | 0.49 | down-regulated |
| Mrpl28 |  | 39S ribosomal protein L28, mitochondrial | 0.02 | 0.22 | down-regulated |
| Rab24 |  | ras-related protein Rab-24 isoform X1 | 0.02 | 0.26 | down-regulated |
| Rp2 |  | protein XRP2 isoform X1 | 0.02 | 0.36 | down-regulated |
| RGD1563986 |  | protein LCHN isoform X1 | 0.02 | 0.42 | down-regulated |
| LOC103690005 |  | tubulin-folding cofactor B | 0.02 | 0.24 | down-regulated |
| Pik3c3 |  | phosphatidylinositol 3-kinase catalytic subunit type 3 isoform X1 | 0.02 | 0.34 | down-regulated |
| Mettl7a |  | methyltransferase-like protein 7A | 0.02 | 0.45 | down-regulated |
| Ubl3 |  | ubiquitin-like protein 3 precursor | 0.02 | 0.32 | down-regulated |
| Grm4 |  | metabotropic glutamate receptor 4 precursor | 0.03 | 0.16 | down-regulated |
| Pvalb |  | parvalbumin alpha isoform X1 | 0.03 | 0.25 | down-regulated |
| Pcdhga3 |  | protocadherin gamma-A3 precursor | 0.03 | 0.12 | down-regulated |
| Smarce1 |  | SWI/SNF-related matrix-associated actin-dependent regulator of chromatin subfamily E member 1 | 0.03 | 0.31 | down-regulated |
| Kank4 |  | KN motif and ankyrin repeat domain-containing protein 4 isoform X2 | 0.03 | 0.46 | down-regulated |
| Mob1b |  | MOB kinase activator 1B | 0.03 | 0.49 | down-regulated |
| Akap10 |  | A-kinase anchor protein 10, mitochondrial isoform X1 | 0.03 | 0.35 | down-regulated |
| Fpgt |  | fucose-1-phosphate guanylyltransferase | 0.04 | 0.31 | down-regulated |
| Acyp2 |  | acylphosphatase-2 | 0.03 | 0.44 | down-regulated |
| Dennd5b |  | DENN domain-containing protein 5B isoform X4 | 0.03 | 0.33 | down-regulated |
| Anapc4 |  | anaphase-promoting complex subunit 4 | 0.03 | 0.19 | down-regulated |
| Iba57 |  | putative transferase CAF17, mitochondrial | 0.03 | 0.44 | down-regulated |
| Ggct |  | gamma-glutamylcyclotransferase | 0.03 | 0.25 | down-regulated |
| Timmdc1 |  | complex I assembly factor TIMMDC1, mitochondrial | 0.04 | 0.06 | down-regulated |
| Srsf4 |  | serine/arginine-rich splicing factor 4 | 0.04 | 0.04 | down-regulated |
| Ubac2 |  | ubiquitin-associated domain-containing protein 2 isoform X1 | 0.04 | 0.07 | down-regulated |
| Rtf1 |  | RNA polymerase-associated protein RTF1 homolog isoform X1 | 0.04 | 0.32 | down-regulated |
| Exoc6 |  | exocyst complex component 6 | 0.04 | 0.49 | down-regulated |
| Pde10a |  | cAMP and cAMP-inhibited cGMP 3',5'-cyclic phosphodiesterase 10A | 0.05 | 0.29 | down-regulated |
| Cdk5rap3 |  | CDK5 regulatory subunit-associated protein 3 | 0.04 | 0.23 | down-regulated |
| Rraga |  | ras-related GTP-binding protein A | 0.04 | 0.46 | down-regulated |
| Mvp |  | major vault protein | 0.04 | 0.18 | down-regulated |
| Ccdc127 |  | coiled-coil domain-containing protein 127 | 0.04 | 0.41 | down-regulated |
| Cd82 |  | CD82 antigen isoform X1 | 0.04 | 0.48 | down-regulated |
| Eif1ad |  | probable RNA-binding protein EIF1AD | 0.05 | 0.29 | down-regulated |
| Lyplal1 |  | lysophospholipase-like protein 1 | 0.05 | 0.01 | down-regulated |
| Daglb |  | sn1-specific diacylglycerol lipase beta isoform X1 | 0.05 | 0.47 | down-regulated |
| Ninj1 |  | ninjurin-1 | 0.05 | 0.28 | down-regulated |
| Herc4 |  | probable E3 ubiquitin-protein ligase HERC4 isoform X3 | 0.03 | 0.55 | down-regulated |
| Lrrc8d |  | volume-regulated anion channel subunit LRRC8D isoform X1 | 0.04 | 0.62 | down-regulated |
| Rexo2 |  | oligoribonuclease, mitochondrial precursor | 0.04 | 0.58 | down-regulated |
| Slc22a23 |  | solute carrier family 22 member 23 | 0.05 | 0.65 | down-regulated |
| NEWGENE_1308105 |  | kinase suppressor of Ras 1 isoform X7 | 0.05 | 0.59 | down-regulated |
| Gan |  | gigaxonin isoform X1 | 0.05 | 0.48 | down-regulated |
| Snx12 |  | sorting nexin-12 isoform X3 | 0.00 | 0.61 | down-regulated |
| Slc6a9 |  | sodium- and chloride-dependent glycine transporter 1 isoform X1 | 0.00 | 0.58 | down-regulated |
| Itch |  | E3 ubiquitin-protein ligase Itchy homolog isoform X1 | 0.00 | 0.51 | down-regulated |
| Pip4k2c |  | phosphatidylinositol 5-phosphate 4-kinase type-2 gamma | 0.01 | 0.65 | down-regulated |
| Phgdh |  | D-3-phosphoglycerate dehydrogenase isoform X1 | 0.01 | 0.63 | down-regulated |
| Pde4d |  | cAMP-specific 3',5'-cyclic phosphodiesterase 4D isoform 3 | 0.01 | 0.57 | down-regulated |
| S100a16 |  | protein S100-A16 isoform X1 | 0.01 | 0.55 | down-regulated |
| Gys1 |  | glycogen [starch] synthase, muscle | 0.01 | 0.53 | down-regulated |
| Acaa1b |  | 3-ketoacyl-CoA thiolase B, peroxisomal precursor | 0.02 | 0.54 | down-regulated |
| Nae1 |  | NEDD8-activating enzyme E1 regulatory subunit | 0.02 | 0.64 | down-regulated |
| Dclk2 |  | serine/threonine-protein kinase DCLK2 isoform X4 | 0.02 | 0.53 | down-regulated |
| Lrrc57 |  | leucine-rich repeat-containing protein 57 isoform X2 | 0.02 | 0.58 | down-regulated |
| Rida |  | 2-iminobutanoate/2-iminopropanoate deaminase | 0.02 | 0.64 | down-regulated |
| Cadm2 |  | cell adhesion molecule 2 precursor | 0.02 | 0.60 | down-regulated |
| Prkcb |  | protein kinase C beta type isoform 1 | 0.02 | 0.67 | down-regulated |
| Gstt3 |  | glutathione S-transferase, theta 3 | 0.02 | 0.64 | down-regulated |
| Dnaja4 |  | dnaJ homolog subfamily A member 4 | 0.03 | 0.63 | down-regulated |
| Efl1 |  | elongation factor-like GTPase 1 | 0.03 | 0.52 | down-regulated |
| RGD1305455 |  | uncharacterized protein C7orf43 homolog | 0.03 | 0.65 | down-regulated |
| Alad |  | delta-aminolevulinic acid dehydratase isoform X1 | 0.03 | 0.64 | down-regulated |
| RGD1564420 |  | WASH complex subunit strumpellin | 0.03 | 0.64 | down-regulated |
| Lnpk |  | protein lunapark isoform X1 | 0.03 | 0.61 | down-regulated |
| Cpne4 |  | copine-4 isoform X1 | 0.04 | 0.56 | down-regulated |
| Nt5c3a |  | cytosolic 5'-nucleotidase 3A isoform X2 | 0.04 | 0.65 | down-regulated |
| Rgs10 |  | regulator of G-protein signaling 10 isoform X1 | 0.05 | 0.66 | down-regulated |
| Mrps30 |  | 28S ribosomal protein S30, mitochondrial | 0.05 | 0.58 | down-regulated |
| Kcnj6 |  | G protein-activated inward rectifier potassium channel 2 | 0.05 | 0.59 | down-regulated |
