# Supplementary material for: Comparative Proteomic Characterization of Ventral Hippocampus in Susceptible and Resilient Rats Subjected to Chronic Unpredictable Stress
Source: Front Neurosci. 2021 Jun 17;15:675430. doi: 10.3389/fnins.2021.675430 (PMC8249003; doi:10.3389/fnins.2021.675430)
Supplement: Supplementary file 4 [file Table_4.docx]

**Table S4. Differentially expressed proteins in stress-susceptible *versus* stress-resilient**.

| Gene symbol |  | Protein Description | *P*-values | Fold change | Significant |
| --- | --- | --- | --- | --- | --- |
| Lias |  | lipoyl synthase, mitochondrial precursor | 0.03 | 2.75 | up-regulated |
| Babam1 |  | BRISC and BRCA1-A complex member 1 isoform X1 | 0.03 | 3.00 | up-regulated |
| Cnnm2 |  | metal transporter CNNM2 | 0.01 | 8.17 | up-regulated |
| Eri3 |  | ERI1 exoribonuclease 3 isoform X8 | 0.05 | 93.69 | up-regulated |
| Jam3 |  | junctional adhesion molecule C precursor | 0.03 | 3.16 | up-regulated |
| Il1rapl1 |  | interleukin-1 receptor accessory protein-like 1 isoform X1 | 0.05 | 11.85 | up-regulated |
| Clec2l |  | C-type lectin domain family 2 member L isoform X1 | 0.01 | 5.09 | up-regulated |
| Arf5 |  | ADP-ribosylation factor 5 | 0.05 | 2.10 | up-regulated |
| Tmem106b |  | transmembrane protein 106B | 0.02 | 2.90 | up-regulated |
| Cnot3 |  | CCR4-NOT transcription complex subunit 3 | 0.04 | 2.64 | up-regulated |
| Hip1 |  | huntingtin-interacting protein 1 isoform X4 | 0.02 | 3.03 | up-regulated |
| Cbarp |  | voltage-dependent calcium channel beta subunit-associated regulatory protein | 0.04 | 2.59 | up-regulated |
| Rab8b |  | ras-related protein Rab-8B | 0.03 | 2.50 | up-regulated |
| Frmd8 |  | FERM domain-containing protein 8 | 0.03 | 9.80 | up-regulated |
| Rnf11l2 |  | RING finger protein 11 | 0.03 | 2.98 | up-regulated |
| Lclat1 |  | lysocardiolipin acyltransferase 1 | 0.02 | 3.25 | up-regulated |
| Gfod1 |  | glucose-fructose oxidoreductase domain-containing protein 1 isoform X1 | 0.02 | 2.26 | up-regulated |
| Timm10 |  | mitochondrial import inner membrane translocase subunit Tim10 isoform X1 | 0.02 | 2.52 | up-regulated |
| Ovca2 |  | esterase OVCA2 | 0.04 | 2.46 | up-regulated |
| Ric8a |  | synembryn-A | 0.02 | 1.58 | up-regulated |
| Pde4d |  | cAMP-specific 3',5'-cyclic phosphodiesterase 4D isoform 3 | 0.00 | 1.77 | up-regulated |
| S100a16 |  | protein S100-A16 isoform X1 | 0.03 | 1.51 | up-regulated |
| Scn1b |  | sodium channel subunit beta-1 isoform 2 | 0.01 | 1.50 | up-regulated |
| Vps37b |  | vacuolar protein sorting-associated protein 37B | 0.01 | 1.71 | up-regulated |
| Pex19 |  | peroxisomal biogenesis factor 19 isoform 2 | 0.01 | 1.55 | up-regulated |
| Ermn |  | ermin | 0.03 | 1.61 | up-regulated |
| Rab22a |  | ras-related protein Rab-22A isoform X2 | 0.03 | 1.60 | up-regulated |
| Pigt |  | GPI transamidase component PIG-T precursor | 0.04 | 1.98 | up-regulated |
| Pde6d |  | retinal rod rhodopsin-sensitive cGMP 3',5'-cyclic phosphodiesterase subunit delta isoform X1 | 0.03 | 1.51 | up-regulated |
| NEWGENE_2116 |  | acidic leucine-rich nuclear phosphoprotein 32 family member A isoform X4 | 0.00 | 0.36 | down-regulated |
| Rpl30l1 |  | 60S ribosomal protein L30-like | 0.03 | 0.47 | down-regulated |
| Sqrdl |  | sulfide:quinone oxidoreductase, mitochondrial isoform X1 | 0.00 | 0.14 | down-regulated |
| Dcun1d3 |  | DCN1-like protein 3 isoform X1 | 0.04 | 0.10 | down-regulated |
| Sidt1 |  | SID1 transmembrane family member 1 isoform X2 | 0.03 | 0.40 | down-regulated |
| Cecr5 |  | cat eye syndrome critical region protein 5 | 0.03 | 0.44 | down-regulated |
| Hars2 |  | probable histidine--tRNA ligase, mitochondrial | 0.03 | 0.05 | down-regulated |
| Prpf3 |  | U4/U6 small nuclear ribonucleoprotein Prp3 isoform X4 | 0.04 | 0.38 | down-regulated |
| Maged1 |  | melanoma-associated antigen D1 | 0.01 | 0.46 | down-regulated |
| Vps41 |  | vacuolar protein sorting-associated protein 41 homolog | 0.00 | 0.14 | down-regulated |
| LOC102556574 |  | serrate RNA effector molecule homolog | 0.02 | 0.01 | down-regulated |
| Dnajc7 |  | dnaJ homolog subfamily C member 7 isoform X2 | 0.00 | 0.38 | down-regulated |
| Atrn |  | attractin precursor | 0.02 | 0.39 | down-regulated |
| Tspyl4 |  | testis-specific Y-encoded-like protein 4 | 0.02 | 0.09 | down-regulated |
| Scamp4 |  | secretory carrier-associated membrane protein 4 isoform X1 | 0.03 | 0.00 | down-regulated |
| Camk2n1 |  | calcium/calmodulin-dependent protein kinase II inhibitor 1 | 0.03 | 0.01 | down-regulated |
| Caap1 |  | caspase activity and apoptosis inhibitor 1 isoform X1 | 0.03 | 0.12 | down-regulated |
| Mavs |  | mitochondrial antiviral-signaling protein isoform X1 | 0.02 | 0.31 | down-regulated |
| Kndc1 |  | protein very KIND isoform X2 | 0.03 | 0.50 | down-regulated |
| Ddx23 |  | probable ATP-dependent RNA helicase DDX23 isoform X1 | 0.03 | 0.44 | down-regulated |
| Plg |  | plasminogen precursor | 0.01 | 0.34 | down-regulated |
| Nup35 |  | nucleoporin NUP53 isoform X1 | 0.02 | 0.11 | down-regulated |
| Ppp1r16a |  | protein phosphatase 1 regulatory subunit 16A | 0.03 | 0.13 | down-regulated |
| Mrps31 |  | 28S ribosomal protein S31, mitochondrial isoform X1 | 0.01 | 0.16 | down-regulated |
| Smc1a |  | structural maintenance of chromosomes protein 1A | 0.03 | 0.26 | down-regulated |
| Pigs |  | GPI transamidase component PIG-S | 0.01 | 0.38 | down-regulated |
| Hsd17b8 |  | estradiol 17-beta-dehydrogenase 8 | 0.03 | 0.48 | down-regulated |
| Cpne3 |  | copine-3 isoform X1 | 0.03 | 0.08 | down-regulated |
| Rnf170 |  | E3 ubiquitin-protein ligase RNF170 | 0.02 | 0.39 | down-regulated |
| Rmdn1 |  | regulator of microtubule dynamics protein 1 isoform X1 | 0.02 | 0.36 | down-regulated |
| Ssbp2 |  | single-stranded DNA-binding protein 2 isoform X7 | 0.04 | 0.08 | down-regulated |
| Dcaf7 |  | DDB1- and CUL4-associated factor 7 | 0.02 | 0.41 | down-regulated |
| Lrsam1 |  | E3 ubiquitin-protein ligase LRSAM1 | 0.00 | 0.19 | down-regulated |
| Dip2a |  | disco-interacting protein 2 homolog A | 0.03 | 0.35 | down-regulated |
| Flnc |  | filamin-C isoform X1 | 0.02 | 0.24 | down-regulated |
| Emc3 |  | ER membrane protein complex subunit 3 | 0.00 | 0.42 | down-regulated |
| Rbp1 |  | retinol-binding protein 1 | 0.04 | 0.36 | down-regulated |
| Ces1c |  | carboxylesterase 1C precursor | 0.03 | 0.23 | down-regulated |
| Gss |  | glutathione synthetase | 0.02 | 0.44 | down-regulated |
| Apaf1 |  | apoptotic protease-activating factor 1 isoform X1 | 0.03 | 0.02 | down-regulated |
| G3bp1 |  | ras GTPase-activating protein-binding protein 1 | 0.00 | 0.44 | down-regulated |
| Park2 |  | E3 ubiquitin-protein ligase parkin | 0.03 | 0.06 | down-regulated |
| Zfand2b |  | AN1-type zinc finger protein 2B | 0.03 | 0.03 | down-regulated |
| Wrb |  | tail-anchored protein insertion receptor WRB | 0.02 | 0.14 | down-regulated |
| LOC108348122 |  | CAP-Gly domain-containing linker protein 3 | 0.02 | 0.24 | down-regulated |
| Cnot2 |  | CCR4-NOT transcription complex subunit 2 isoform X6 | 0.03 | 0.43 | down-regulated |
| Serinc1 |  | serine incorporator 1 precursor | 0.03 | 0.08 | down-regulated |
| Eif2ak2 |  | interferon-induced, double-stranded RNA-activated protein kinase isoform X2 | 0.01 | 0.27 | down-regulated |
| Cdh9 |  | cadherin-9 isoform X1 | 0.01 | 0.26 | down-regulated |
| Ccm2 |  | cerebral cavernous malformations 2 protein isoform X4 | 0.02 | 0.41 | down-regulated |
| Dtnb |  | dystrobrevin beta isoform X1 | 0.04 | 0.49 | down-regulated |
| Slc5a3 |  | sodium/myo-inositol cotransporter | 0.03 | 0.53 | down-regulated |
| Gigyf2 |  | PERQ amino acid-rich with GYF domain-containing protein 2 isoform X3 | 0.05 | 0.53 | down-regulated |
| Gaa |  | lysosomal alpha-glucosidase isoform X1 | 0.00 | 0.57 | down-regulated |
| Rcn1 |  | reticulocalbin-1 precursor | 0.01 | 0.57 | down-regulated |
| Copg1 |  | coatomer subunit gamma-1 | 0.04 | 0.63 | down-regulated |
| Plbd2 |  | putative phospholipase B-like 2 isoform X1 | 0.04 | 0.63 | down-regulated |
| Nav1 |  | neuron navigator 1 isoform X5 | 0.02 | 0.57 | down-regulated |
| Cmbl |  | carboxymethylenebutenolidase homolog isoform X1 | 0.00 | 0.54 | down-regulated |
| Eif3k |  | eukaryotic translation initiation factor 3 subunit K isoform X1 | 0.02 | 0.52 | down-regulated |
| Gopc |  | Golgi-associated PDZ and coiled-coil motif-containing protein | 0.04 | 0.54 | down-regulated |
| H2afz |  | histone H2A.Z | 0.01 | 0.60 | down-regulated |
| Phka1 |  | phosphorylase b kinase regulatory subunit alpha, skeletal muscle isoform isoform X2 | 0.01 | 0.58 | down-regulated |
| Gga3 |  | ADP-ribosylation factor-binding protein GGA3 isoform X1 | 0.00 | 0.66 | down-regulated |
| Entpd2 |  | ectonucleoside triphosphate diphosphohydrolase 2 precursor | 0.03 | 0.66 | down-regulated |
| Asphd2 |  | aspartate beta-hydroxylase domain-containing protein 2 isoform X1 | 0.04 | 0.53 | down-regulated |
