# Supplementary material for: Comparative Proteomic Characterization of Ventral Hippocampus in Susceptible and Resilient Rats Subjected to Chronic Unpredictable Stress
Source: Front Neurosci. 2021 Jun 17;15:675430. doi: 10.3389/fnins.2021.675430 (PMC8249003; doi:10.3389/fnins.2021.675430)
Supplement: Supplementary file 5 [file Table_5.docx]

**Supplementary material**

**1. Chronic unpredictable stress procedure**

All stressors applied were shown in Table S1.

**2. Behavioral tests procedure**

**2.1 Sucrose preference test**

Sucrose preference test was employed to reflect the lack of pleasure in animals, which is the main pathological feature of depression. Briefly, each rat was placed in a separate cage, all rats were preconditioned with 1 % sucrose solution for 48 h, and then were water deprived for 24 h. After that, each rat was given two same-shaped bottles, one bottle had 1% sucrose solution, another bottle had water. Location of two drinking bottles were exchanged arbitrarily. All bottles were weighed before and after the test. Sucrose preference (%) = (sucrose intakes) / (sucrose intakes + water intakes) × 100 %.

**2.2 Forced swimming test**

Forced swimming test was performed in a transparent swim cylinder, the diameter of the swim cylinder was 20 cm, the water depth was 60 cm, and water temperature was kept at 23 ± 1℃. FST protocol was divided into two sessions. First stage was the behavior training. Each rat was put into the water one by one for 5 minutes and then returned to their cages for normal breeding. After 24 hours, formal behavior tests were started. The immobility time was recorded within 6 minutes. After the experiment, the rats were immediately dried, and returned to their cages. The immobility time was an indicator of the animal's desperate and helpless behavior.

**2.3 Open field test**

Open field test (OFT) was conducted in a resin-coated white square box (100 cm × 100 cm ×40 cm). The bottom of the box was divided into 25 same-size squares by black lines. Before the OFT treatment, each rat was acclimated in the box for 5 minutes. At the beginning of the experiment, the rat was placed in the central area of the box, its free exploration activities were recorded and timed by the camera for 5 minutes. After OFT, the box was immediately cleaned with 50% alcohol to avoid disturbances of secretions and odors to the next experiment. The number of crossings and rearing of hind limbs of the rat within 5 minutes were employed to assess autonomous movement ability.

Table S1. Chronic unpredictable stress procedure

| Day | Stressors |
| --- | --- |
| Day1 | Swim stress 10 ℃, 5min; cage tilt 45° overnight |
| Day2 | Restraint 4 h; reversal of the light/dark cycle (light off 12 h and light on overnight) |
| Day3 | Electric shock, 3 min; shaker stress 2 h |
| Day4 | Tail pinch 1 min; food deprivation 24 h |
| Day5 | Isolation 48 h; water deprivation 24 h |
| Day6 | Ultrasonic stress 2 h; isolation 24 h |
| Day7 | Crowding overnight (8 rats per cage); wet bedding overnight (200 mL water spilled onto 100 g sawdust bedding) |
